# Face Your Fears: Virtual reality-based cognitive behavioral therapy (VR-CBT) versus standard CBT for paranoid ideations in patients with schizophrenia spectrum disorders: a randomized clinical trial

**DOI:** 10.1186/s13063-022-06614-0

**Published:** 2022-08-15

**Authors:** U. N. Jeppesen, A. S. Due, L. Mariegaard, A. Pinkham, M. Vos, W. Veling, M. Nordentoft, L. B. Glenthøj

**Affiliations:** 1 Copenhagen Research Centre on Mental Health (CORE), University of Copenhagen, Copenhagen, Denmark; 2 Department of Psychology, University of Copenhagen, Copenhagen, Denmark; 3 School of Behavioral and Brain Sciences, University of Texas at Dallas, Richardson, USA; 4 Faculty of Medical Sciences, University Medical Center Groningen, Center of Psychiatry, University of Groningen, Groningen, Netherlands

**Keywords:** Schizophrenia spectrum disorders, Schizotypal disorders, Delusions, Paranoid ideations, Ideas of reference, Cognitive behavioral therapy, Virtual reality exposure therapy, Virtual reality, Social functioning, Activities of daily living

## Abstract

**Background:**

Schizophrenia spectrum disorders cause suffering for patients, relatives, and the surrounding society. Paranoid ideations, encompassing ideas of social reference and manifest persecutory delusions, are among the most frequent symptoms in this population and a cause of significant distress. Recent meta-analyses of cognitive behavioral therapy (CBT) for psychosis show small to moderate effect sizes in reducing paranoid ideations. Virtual reality-based CBT (VR-CBT) could improve therapy efficacy as exposure and behavioral experiments in VR can be optimized, individualized, and carried out in a safe environment. Few VR-CBT studies exist for paranoid ideations and there is a need for large-scale, methodologically rigorous trials.

**Methods:**

This study is a randomized, assessor-blinded parallel-groups multi-center superiority clinical trial, fulfilling the CONSORT criteria for non-pharmacological treatment. A total of 256 patients diagnosed with schizophrenia spectrum disorder, including schizotypal disorder (ICD-10 F20-29), will be allocated to either 10 sessions of symptom-specific CBT-VR plus treatment as usual—versus 10 sessions of standard symptom-specific CBT for paranoid ideations (CBT) plus treatment as usual. All participants will be assessed at baseline, treatment end (3 months post baseline), and then 9 months post baseline. A stratified block-randomization with concealed randomization sequence will be conducted. Independent assessors blinded to the treatment will evaluate the outcome. Analysis of outcome will be carried out with the intention to treat principles.

The primary outcome is ideas of social reference measured with Green Paranoid Thought Scale Part A (GPTS-A) at the cessation of treatment at 3 months post baseline. Secondary outcomes are ideas of persecution (GPTS-B), Social Interaction Anxiety Scale (SIAS), Personal and Social Performance scale (PSP), Safety Behavior Questionnaire (SBQ), and CANTAB Emotion Recognition Task.

**Discussion:**

The trial will elucidate whether VR-CBT can enhance therapy efficacy for paranoid ideations. Additionally, Trial findings will provide evidence on the effectiveness and cost-effectiveness of VR-CBT for paranoid ideations that can guide the possible dissemination and implementation into clinical practice.

**Trial registration:**

ClinicalTrials.govNCT04902066. Initial release April 9th, 2021.

## Administrative information

Note: the numbers in curly brackets in this protocol refer to SPIRIT checklist item numbers. The order of the items has been modified to group similar items (see http://www.equator-network.org/reporting-guidelines/spirit-2013-statement-defining-standard-protocol-items-for-clinical-trials/).

**Table:** 

Title {1}	Face Your Fears: Virtual reality-based cognitive behavioral therapy (VR-CBT) versus standard CBT for paranoid ideations in patients with schizophrenia and related disorders: A randomized clinical trial.
Trial registration {2a & 2b}	ClinicalTrials.gov Identifier: NCT04902066. Initial release April 9th, 2021. https://www.clinicaltrials.gov/ct2/show/NCT04902066
Protocol version {3}	Study protocol, revised version for submission, 25th of July, 2022.
Funding {4}	Trygfoundation (ID: 148727) Independent Research Fund Denmark (0134-00066B) Research Fund of the Mental Health Services – Capital Region of Denmark Research Fund for Health Research 2019 – Capitol Region of Denmark (A6622)
Author details {5a}	1: Copenhagen Research Centre on Mental Health (CORE), University of Copenhagen (DK) 2: University of Copenhagen, Department of Psychology (DK) 3: University of Texas at Dallas, School of Behavioral and Brain Sciences (US) 4: University of Groningen, Faculty of Medical Sciences, University Medical Center Groningen, Center of Psychiatry (NL)
Name and contact information for the trial sponsor {5b}	Trygfoundation, Hummeltoftevej 49, 2830 Virum, Denmark. Phone +45 45260800. info@trygfonden.dk Independent Research Fund Denmark, Asylgade 7, 5000 Odense C, Denmark, Phone +45 35446800, dff@ufm.dk Research Fund of the Mental Health Services – Capital Region of Denmark, Kongens Vænge 2, 3400 Hillerød, Denmark, Phone +45 38665000, pure@regionh.dk Research Fund for Health Research – Capitol Region of Denmark, Kongens Vænge 2, 3400 Hillerød, Denmark, Phone +45 38665000, pure@regionh.dk
Role of sponsor {5c}	The sponsor played no part in study design; collection, management, analysis, and interpretation of data; writing of the report; and the decision to submit the report for publication.

## Introduction

### Background and rationale {6a}

Schizophrenia spectrum disorders (ICD-10, F20-29) commonly have an onset in young age; in many cases, have long-term consequences; and cause suffering on both patients and relatives, as well as society at large [1]. Schizophrenia prevalence is roughly consistent worldwide with a prevalence of approximately 0.3% and it has been stable over the last decades [2]. The associated costs of the disorder for the individual is profound. This is reflected in the fact that schizophrenia has one of the highest measured Years Lived with Disability (YLDs) among all diseases, injuries, and risk factors [2].

Paranoid ideations, encompassing ideas of social reference and manifest persecutory delusions [3], are common symptoms in schizophrenia spectrum disorders [4, 5]. Social reference refers to experiences of reference with an observational theme when meeting or engaging with other people, e.g., a sense of an always intentional, peculiar, negative gaze (being seen through and exposed, criticized, mocked, condescended), gossiping, and detailed observations of one’s actions from others. It is believed that there is a spectrum or hierarchy [6, 7] of paranoid ideations from this passive attitude of others to persecutory experiences where the intention of others is perceived as actively trying to harm. In more severe psychopathological conditions, i.e., states of delusion, persecutory experiences tend to be most prominent. While a large group of patients with first episode psychosis achieves remission of psychotic symptoms during the first year after initial contact with mental health services, almost one-third still have psychotic symptoms, in spite of treatment with antipsychotic medication [8–10]. The social avoidance caused by paranoid ideations does not improve with antipsychotic medication and the unfounded threat beliefs of other people are maintained [11]. Paranoid ideations and the associated social avoidance therefore have severe consequences for the patients’ ability to interact with other people and often prevent them from conducting basic daily activities—as well as reducing their quality of life [12–14].

Cognitive behavioral therapy for psychosis (CBT) aims primarily at treating delusions and other psychotic symptoms. During the last decades, there has been a renewed interest in CBT for psychosis leading to an increased number of randomized clinical trials (RCTs) [15–18]. Accumulating evidence in recent years reports CBT to have stable effect sizes in the small to moderate range in reducing paranoid ideations [17, 18]. Adopting a more targeted treatment approach has improved outcomes. Such a targeted approach comprises symptom-specific treatment protocols which have effect sizes closer to the moderate range [19]. Symptom specific can be defined as: The symptom of interest is present at a substantial level; it is the primary target in therapy and aspects of the symptom are the outcome measures of interest [19]. Besides a more targeted approach, the appropriate weighting between the behavioral and cognitive components of therapy is important, as meta-analytical evidence shows that CBT for paranoid ideations is significantly better when focusing on the behavioral component of therapy [20]. The behavioral component is however challenging to conduct as exposure in a real-life setting eliciting paranoid ideations is difficult to organize and control.

Virtual Reality (VR) exposure cannot only be symptom-specific but also improve the behavioral component of therapy, as it provides control over, safety in, and fast access to relevant situations in a virtual environment, that are created for specific symptoms and common situations they often occur in. It allows possibilities for individualizing treatment and thereby achieving the desirable level of arousal in each situation.

Research on virtual reality-based cognitive behavioral therapy (VR-CBT) targeting psychotic symptoms is still in its infancy. Only two studies have investigated the effectiveness of therapist-guided VR-CBT for paranoid ideations in patients with a schizophrenia spectrum disorder: Freeman et al. (2016) [21] conducted a pilot study on the effect of VR cognitive therapy versus VR exposure for 30 patients with persecutory delusions. The pilot study found brief VR cognitive therapy (7 sessions) to lead to large reductions in delusional conviction (Cohen’s *d* = 1.3) and real-world distress (Cohen’s *d* = 0.8), compared to virtual reality exposure. The study was, however, a pilot study and did suffer important methodological limitations such as using non-blinding of assessors and a limited post-treatment assessment period without follow-up. The second study by Pot-Kolder et al. [22] compared the effectiveness of VR-based cognitive behavioral therapy (VR-CBT) versus waiting list control in 116 patients with psychosis experiencing paranoid ideations and social avoidance. The study found 16 sessions of VR-CBT to be superior to treatment as usual (TAU), i.e., waiting list, in reducing the level of persecutory delusions as measured by the Green Paranoid Thought Scale (GPTS) [22]. This was a secondary outcome. Posttreatment effect size was moderate to large (Cohen’s *D* = 0.44 for ideas of social reference and 0.7 for ideas of persecution) and increased at 6 months follow-up (Cohen’s *d* = 0.55 for ideas of reference and 0.78 for ideas of persecution). The study though lacked an active control group. Additionally, this study provided important short-term results of cost-effectiveness [23]

Consequently, to establish an evidence base for the efficacy of VR-CBT there is a need for well-controlled and adequately powered studies comparing VR-CBT with standard, symptom-specific CBT, with the latter defined as the current psychotherapeutical gold standard. Hence, there is a clear need for large-scale, methodologically rigorous trials testing the effectiveness of VR-CBT on multiple outcomes along with its cost-effectiveness. This may guide the potential of dissemination and implementation of VR-CBT into clinical practice [23].

It can be argued that VR-CBT may be useful in several stages of disease development and treatment: that is first episode psychosis and treatment-resistant schizophrenia and additionally as an add-on to antipsychotic medication or a stand-alone treatment [24]. Furthermore, it may also be applied to patients with schizotypal conditions, experiencing ideas of social reference and persecution. In these conditions, evidence for antipsychotic medication is weak [25] and more psychotherapeutic studies in general are needed [26].

If the efficacy of CBT for psychotic symptoms could be further improved by augmenting it with VR, it could be an important step in transferring CBT for psychosis from research trials to real-life clinical settings on a significant scale with guidance in the possible dissemination and implementation.

### Objectives {7}

We hypothesize that:

- VR-CBT will be superior to CBT in reducing ideas of social reference in patients with schizophrenia spectrum disorders (ICD-10, F20-29, schizotypal disorder included).
- VR-CBT will be superior to CBT in reducing ideas of persecution, social anxiety, avoidance, and safety behavior, as well as improving social cognition, psychosocial functioning, and quality of life in patients with schizophrenia spectrum disorders (ICD-10, F20-29, schizotypal disorder included).

### Trial design {8}

The study is a high-quality randomized, assessor-blinded parallel-groups superiority clinical trial fulfilling the CONSORT criteria for non-pharmacological treatment. A total of 256 patients will be allocated to either VR-CBT plus TAU or CBT plus TAU (see flowchart below). All participants will be assessed at baseline, treatment end (3 months post baseline), and then 9 months post baseline. A stratified block-randomization with concealed randomization sequence will be conducted. Independent assessors blinded to the treatment allocation will evaluate the outcome. Analysis of outcome will be carried out with the intention to treat principles.

## Methods: participants, interventions, and outcome

### Study setting {9}

A multicenter study where recruitment will be from outpatient routine care setting for patients suffering from schizophrenia spectrum disorders in the Capital Region of Denmark and the North Denmark Region, i.e., early intervention (OPUS) and Flexible Assertive Community Treatment (F-ACT) facilities. The randomized clinical trial will be conducted in the university hospitals at Mental Health Center Copenhagen [27] and Aalborg university hospital [28].

### Eligibility criteria {10}

The GPTS [3] will be used to assess paranoid ideations with a cut-off of ≥40 on the total score similar to other studies [22, 29]. This is assumed as a threshold for a milder degree of ideations and corresponds to -2sd’s from the mean in a clinical sample [3] (Tables 1 and 2).

**Table 1:** Inclusion criteria

1. ≥ Age 18 years 2. Ability to give informed consent 3. A schizophrenia spectrum disorder (ICD-10 code: F20 -F29, schizotypal disorder included) 4. GPTS total score ≥ 40

**Table 2:** Exclusion criteria

1. A diagnosis of organic brain disease 2. IQ of 70 or lower (known mental retardation as assessed by medical record) 3. A command of spoken Danish or English inadequate for engaging in therapy assessed at baseline interview 4. Inability to tolerate the assessment process	

### Who will take informed consent? {26a}

Trained medical doctors and psychologists are conducting trial assessments and they introduce the trial for referred patients. Information sheets, written in layman’s language, are handed out beforehand in the outpatient routine care setting. Clinicians managing the patient’s TAU in the outpatient facilities are also introduced to and informed about the project through ongoing visits and presentations. Trial assessors will discuss the trial with referred patients considering the information provided in the information sheet and patients’ former discussions with clinicians managing the patient’s TAU in outpatient routine care settings. Assessors will obtain written consent from patients willing to participate in the trial. Decisional capacity is implicitly assessed and approved by psychiatrists in charge of TAU in the outpatient facilities when they refer patients. This is further ongoingly assessed during the trial.

### Additional consent provisions for collection and use of participant data and biological specimens in ancillary studies {26b}

N/A; no ancillary studies planned at this point of time. There are no biological samples.

## Interventions

### Explanation for the choice of comparators {6b}

VR-CBT may, as a novel and promising psychotherapeutical intervention, improve effect size in treating paranoid ideation even further compared to more thoroughly researched psychotherapeutical interventions, and a head-to-head comparison with an active control group is needed to clarify this question [22]. VR-CBT is compared with an active control group who receives standard symptom-specific CBT defined as the current psychotherapeutical gold standard. This control comparator is assumed to be highly appropriate and beneficial for participants as the effect size in treating paranoid ideations may be close to moderate [19] and participants do not receive this as standard treatment in their TAU outpatient routine care settings.

### Intervention description {11a}

Patients in both conditions will be offered 10 individual sessions. 10 sessions have been chosen on the basis of study settings in a former research study [22] and the clinical setting in the Danish Public Healthcare system. One treatment group will receive CBT augmented with virtual reality (VR-CBT), while the other group will receive standard, symptom-specific CBT for psychosis. The treatment will be conducted by psychologists that will receive specific training in conducting the CBT and VR-CBT treatment. Both treatment conditions will be manualized, and the therapists will receive ongoing supervision by an international psychologist expert in CBT and VR-CBT for paranoia [30].

#### VR-CBT (experimental group)

The VR-CBT consists of traditional symptom-specific CBT, described in detail below, with the augmentation of virtual reality exposure [22]. The virtual reality exposure comprises virtual social environments (a bus, café, street, park, and supermarket). These are daily social situations that generally elicit paranoid ideations in patients with schizophrenia spectrum disorders. While virtually engaging in these distressing situations, the therapist will facilitate a CBT dialog aimed at generating alternative (i.e., non-threatening) thinking, diminishing safety behaviors (e.g., avoiding eye gaze), and building up new coping strategies. This is expected to alleviate distress and anxiety and improve daily social functioning. The virtual reality exposure will be by use of the Social World software (www.clevr.net), which allows for a fine-tuning and individualization of the therapy; that is for example by varying the number of avatars situated in the virtual social environment chosen from a large catalog of avatars with different features as age, gender, clothing and ethnicity and varying the avatars response to the patient (e.g., by making the avatar show neutral, friendly or hostile body language and varying facial expressions and duration of eye contact). The VR program also allows for the avatars to say pre-recorded sentences and the therapist to talk with a transformed voice through an avatar making roleplay more realistic. The same situation can be role-played repeatedly allowing the patient to generate alternative responses or actions. Worst-case scenarios can be brought to life giving patients an immense feeling of empowerment in less severe real-life situations. This virtual exposure allows for a controlled environment in which the patients can be confronted with feared social situations in a personalized and safe manner. Preliminary findings reveal this virtual reality program to be well-tolerated and highly effective in reducing paranoid ideations and anxiety in psychosis [22].

#### CBT (control group)

The treatment in the CBT group will follow the core principles of CBT used for psychotic disorders and more specifically on treating paranoid ideations. The CBT treatment facilitates an individualized, problem-oriented approach, and uses techniques such as developing a problem and goal list, normalizing psychotic-like experiences, psychoeducation, working with dysfunctional core beliefs, evaluation of appraisals, probability calculation, and removing or diminishing safety behavior [24]. Real-life exposure and behavioral experiments will be practiced as both homework and in session with the therapist present as far as possible.

#### Fidelity

All therapy sessions will be audiotaped and a number of 7 therapy sessions in both treatment groups will be rated by an independent, experienced CBT psychologist for treatment fidelity using the Cognitive Therapy Rating Scale [31].

### Criteria for discontinuing or modifying allocated interventions {11b}

Treatment and post-baseline assessments may be postponed if participants are hospitalized during the period of treatment and therefore are unable to complete therapy within the designated trial period. Any psychiatric admission and number of hospitalization days will be mapped upon trial completion.

### Strategies to improve adherence to interventions {11c}

Therapists are in ongoing dialog with participants during the therapeutical intervention, and improvement of adherence is considered implicit in this dialog. Therapists can further contact the clinicians managing the patient’s TAU in the outpatient routine care setting who continue to have contact with participants during the intervention and therefore can help strengthen adherence.

### Relevant concomitant care permitted or prohibited during the trial {11d}

#### Medication

The trial does not involve the treatment of the patients with antipsychotic medication. Trial participants will receive the medication prescribed by the treatment responsible psychiatrist managing their treatment in OPUS or F-ACT and it is therefore permitted during the trial. At ascertainment, we aim for no scheduled changes in antipsychotic medication within the 12-week treatment period. Records of participants’ medication will be mapped upon trial completion.

#### Psychosocial intervention

We aim for no other scheduled concomitant psychosocial intervention taking place during the 12-week treatment period, but these are permitted during the trial. Records of participants’ potential concomitant psychosocial intervention during the trial will be mapped upon trial completion.

### Provisions for post-trial care {30}

Outpatient routine care settings in the Danish Public Healthcare system have the formal treatment responsibility before, during, and after the trial intervention, as trial intervention is considered an add-on treatment to TAU, and appropriate post-trial care will thereby be provided in national standards.

### Outcomes {12}

#### Primary outcome

- Level of ideas of social reference is measured with the GPTS ideas of social reference subscale at the cessation of treatment at 3 months. The GPTS is a self-report and has displayed good reliability and validity in patients with psychosis and has also been used in patients at-risk for psychosis showing subthreshold psychotic symptoms (i.e., patients with schizotypal-like symptomatology) [3, 22, 32].

#### Secondary outcomes

- Level of ideas of persecution is measured with the GPTS ideas of persecution subscale [3]
- Level of social interaction anxiety is self-reported with the Social Interaction Anxiety Scale (SIAS) [33, 34]
- Level of global daily life functioning is measured with the semi-structured interview Personal and Social Performance scale (PSP) [35]
- Level of avoidance and safety behavior is measured with the semi-structured interview Safety Behavior Questionnaire (SBQ) [36]
- Ability to recognize emotions is measured with the CANTAB Emotion Recognition Task (ERT) [37].

#### Exploratory outcomes

- Cost-effectiveness analysis will be conducted by using the self-report EQ-5D-5L [38]
- Level of quality of life is measured with the self-report WHO five [39]
- Level of positive symptoms is measured with the semi-structured interview Scale for the Assessment of Positive Symptoms (SAPS) [40]
- Level of negative symptoms is measured with the semi-structured interview the Brief Negative Symptoms Scale (BNSS) [41]
- Level of depressive symptoms is measured with the semi-structures interview Calgary depression scale (CDSS) [42]
- Level of suicidal ideations are measured with the self-report Suicidal Ideation Attributes Scale (SIDAS) [43]
- Level of subjective subtle basic symptoms is measured with the semi-structured interview Cognitive Disturbances scale (COGDIS) [44]
- Level of cognitive tendencies and subjective social cognitive impairments are measured with the self-report Davos Assessment of Cognitive Biases Scale (DACOBS) [45]
- Level of self-efficacy is measured with the self-report of the General Self-Efficacy Scale [46]
- Attributional bias is measured with the Intentionality Bias Task (IBT) [47] and the Trustworthiness scale [48]
- Performance-based social skills functional capacity is measured with the semi-structured roleplay Social Skills Performance Assessment (SSPA) [49]
- Personality traits are measured with the self-report Big five [50]
- The extent and types of traumatic life events are measured with the self-report Trauma And Life Events checklist (TALE) [51]
- A sensitivity analysis will be conducted comparing GPTS scores and the Revised GPTS scores (R-GPTS) [52]
- Level of treatment satisfaction is measured with the self-report Client Satisfaction Questionnaire (CSQ) [53]

### Participant timeline

**Fig. 1:**
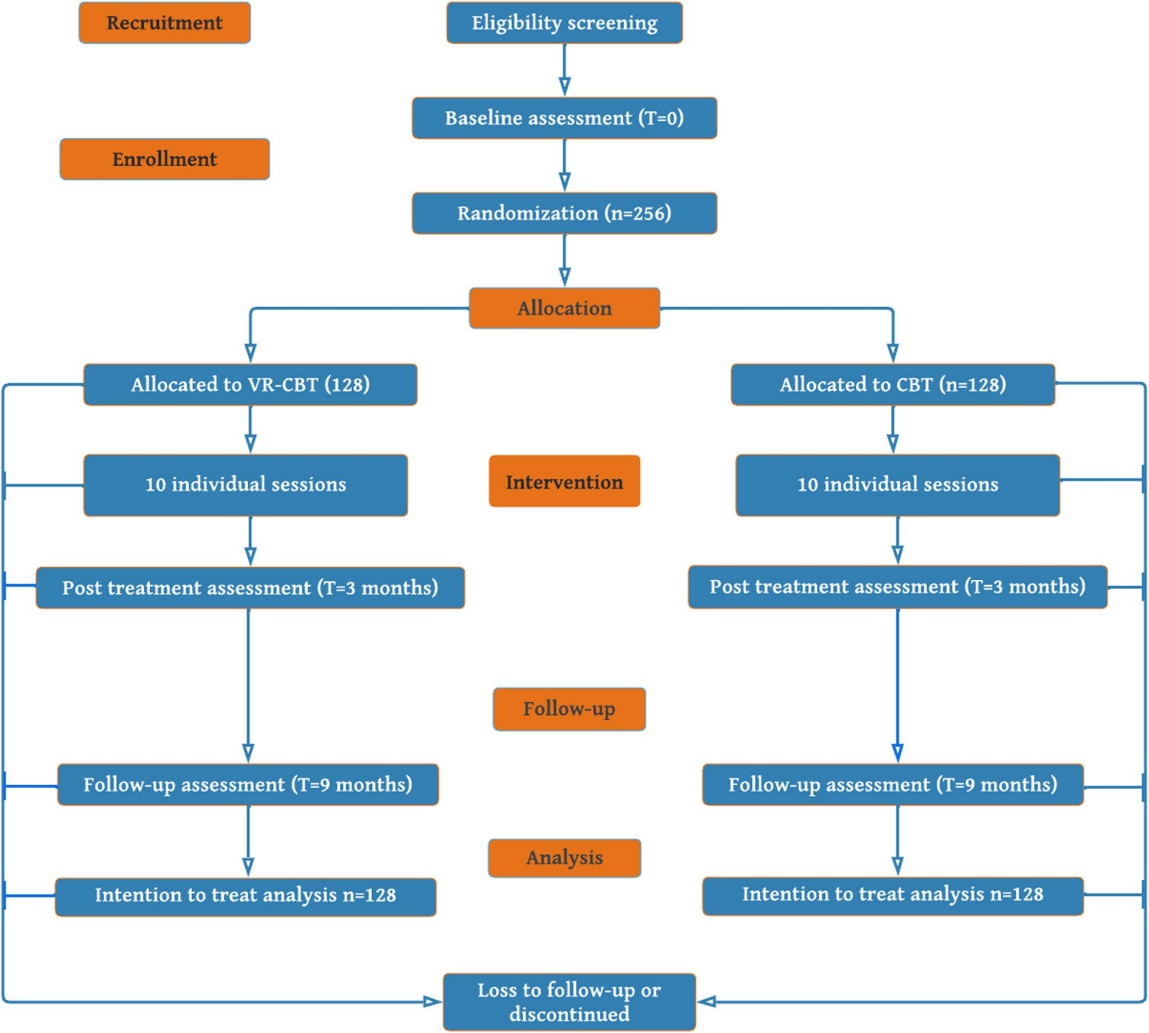
Flow of participants

The participant timeline is shown in Fig. 1.

### Sample size {14}

Sample size calculation is based on our primary outcome, a priori defined to be the between-group difference at 3 months on the GPTS ideas of social reference subscale. We consider a clinically relevant between-group difference on this scale to be 5.5 corresponding to an effect size of Cohen’s *D* = 0.33 (with a pooled SD of 15.5) [22]. With a two-sided alpha of 0.05 and a power of 81%, this will require 128 participants to be randomized to each of the two intervention arms (i.e., 256 participants in total).

### Recruitment {15}

The study will enroll participants from the outpatient routine care facilities in the Capital Region of Denmark and the North Denmark Region (early intervention services (OPUS teams) or Flexible Assertive Community Treatment teams (F-ACT teams)). Patients meeting the listed criteria will be approached about participating in this clinical trial. To avoid unintentional recruitment bias, assessors will regularly attend conferences at the recruitment sites and present the trial as well as invite clinicians to discuss potential referrals.

## Methods: assignment of interventions

### Sequence generation {16a}

Patients providing written informed consent and fulfilling criteria for participation after baseline assessment are allocated to either CBT-VR or CBT. Randomization is performed by a centralized, concealed computer-generated randomization system in REDCap (see Data management section). Block size is created by an independent statistician and will be unknown to the trial assessors and clinicians in the outpatient routine care facilities. The randomized intervention allocation is concealed until the statistical analyses of the resulting data have been completed. Randomization will be stratified by gender, study site, and symptom severity of paranoid ideation (dichotomized as a GPTS Part B score of ≥45 or <45 [3]).

### Concealment mechanism {16b}

Trial assessors write to a specific e-mail when participants have finished baseline assessment and are ready for randomization and treatment allocation. Assessors receive an informatory e-mail, confirming that their e-mail has been received (closed loop communication). One of the therapists has been given the task of performing the allocation sequence and has exclusive access to the randomization program in REDCap (see the “Data management” section), and treatment allocation is thereby concealed for assessors who remain blinded.

### Implementation {16c}

Allocation sequences are generated by the abovementioned therapist with extended access in REDCap at each study site. Enrolment of participants and the intervention assignment will be performed by the therapist who will oversee the given therapeutical course.

## Assignment of interventions: Blinding

### Who will be blinded? {17a}

Independent investigators will conduct the assessments. These trial assessors will be blinded to the treatment allocation of the participants. Due to the nature of the intervention, participants, therapists performing the treatment, and clinicians in charge of TAU will not be blinded to the treatment received. Blinds are maintained by physically separating assessors from therapists performing the treatment. Participants will be instructed not to disclose their allocation prior to assessments continuously through the trial, both orally and in writing. In case an assessor is unblinded, the assessment will be conducted by another blinded trial assessor.

### Procedure for unblinding if needed {17b}

Emergency unblinding of trial assessors is not considered necessary as there is continuous communication between participants, trial therapists, and clinicians in charge of TAU in routine care settings during the trial intervention.

## Methods: Data collection and management

### Plans for assessment and collection of outcomes {18a}

Assessment will be conducted at baseline, treatment end (3 months post baseline), and then 9 months post baseline. To limit the time required to complete the assessments, 5–6 surveys, depending on whether it is baseline or follow-up, will be digitally sent to the participant prior to each assessment with instructions to complete the assessments before the scheduled visit.

The assessors will be psychologists or medical doctors. All assessors will receive adequate training prior to conducting the assessments. The assessors will attend approximately monthly inter-rater reliability training on key measures in the battery and supervised sparring will be performed on weekly basis.

### Plans to promote participant retention and complete follow-up {18b}

Patients participating in the study will be contacted by telephone one week prior to the planned follow-up assessments. Furthermore, clinicians managing the patient’s TAU can help motivate participants to complete follow-up assessments. Assessors are flexible and can rearrange the scheduled time if needed. In case a participant meets discontinuation or exclusion criteria during the study or leaves the intervention program prematurely, clinical and functional assessments will still be performed, if possible.

### Data management {19}

The assessors will enter data from the patient interview directly into the electronic CRF (Case Report Form) using the data entry system REDCap. REDCap is an electronic data capture tool hosted at CIMT in the Capital Region of Denmark [54]. When necessary, the collection will be done in paper and later entered electronically. In REDCap, surveys can be digitally sent to E-boks and the completed form can be returned digitally reducing risk of data loss and leak. E-boks is a public Danish mailing system all inhabitants are in possession of where person sensitive information can be sent safety. REDCap has a complete audit trail on all data transactions, detailed user rights and access control management and thereby complies with Danish legislation (Databeskyttelsesforordningen). Data for each patient is connected to a unique serial number. Only assigned researchers can access REDCap with all the data. Data on paper is stored locally and secured. Research data will be exported from REDCap without personal identifiers. Data will be exported to all well-known software packages: (SPSS, SAS, Stata, R.) and put in logged folders on a network drive under the control of the Capital Region of Denmark, CIMT [54]. A data manager will ensure that all variables are properly defined with variable and value labels. All derived variables will be properly defined, and algorithms will be kept in special files. All data will be examined carefully to identify errors in data entry.

### Confidentiality {27}

Communication between trial researchers and clinicians in charge of TAU in the outpatient routine care settings takes place through a secured e-mail system used elsewhere by clinicians in the Danish Public Healthcare System when communicating person-sensitive information. Collection and maintenance of personal information are described in the abovementioned Data management section.

### Plans for collection, laboratory evaluation, and storage of biological specimens for genetic or molecular analysis in this trial/future use {33}

N/A; no biological specimens were collected as part of this trial.

## Statistical methods

### Analysis of outcomes {20a}

The planned comparisons between the two groups on continuous outcomes will be carried out with a generalized linear model adjusted for stratification variables, potential baseline imbalances, and skewed attrition, with missing data handled by multiple imputations. As an important secondary assessment of this type of outcome, linear mixed model analyses with repeated measurements and an unstructured covariance matrix will assess the interaction term between time and intervention. All analyses will be according to the intention-to-treat principle, analyzing all participants in the groups they were assigned to by randomization; that is all participants irrespective of whether they attended follow-up assessments or not. Primary efficacy analyses will be conducted by a blinded and independent statistician that has no contact with participants in the trial.

### Interim analyses {21b}

N/A; no interim analyses are conducted in this trial.

### Methods for additional analyses (e.g., subgroup analyses) {20b}

N/A; no subgroup analyses are conducted in this trial.

### Methods in analysis to handle protocol non-adherence and any statistical methods to handle missing data {20c}

We expect to encounter missing data, and this will be handled with the linear mixed models and multiple imputations as appropriate.

### Plans to give access to the full protocol, participant level-data and statistical code {31c}

No later than 5 years after finishing the last 9 months post baseline interview, we will deliver a completely deidentified data set to an appropriate data archive for sharing purposes.

## Oversight and monitoring

### Composition of the coordinating center and trial steering committee {5d}

The trial will be overseen by a Project Management Group (PMG). The PMG will be comprised of the Primary Investigator (PI) of the study, the daily leader of the trial, the principal therapist of the trial and representatives from each of the sites conducting the study, i.e., the Capital Region of Denmark and the North Denmark Region. The PMG will have a videoconference approximately every month during the recruitment period.

### Composition of the data monitoring committee, its role, and reporting structure {21a}

As the trial has minimal risks, there will be no Data Monitoring Committee (DMC).

### Adverse event reporting and harms {22}

The only known common side-effect of VR is cybersickness, which can be compared to common motion sickness. If encountered, it typically resolves after only a few VR exposures as tolerance occurs. Psychotherapy in VR is generally considered well-tolerated [55]. Hence, we do not expect any serious adverse events to occur. The listed possible serious adverse events are monitored throughout the study: (1) hospital admissions, (2) suicide attempts, 3) any violent incidents necessitating police involvement (whether victim or accused), (4) self-harming behavior, and (5) all deaths.

### Frequency and plans for auditing trial conduct {23}

The PI of this study is a member of the PMG thus making sure information regarding trial conduct is shared across groups. Thus, at least on a monthly basis information regarding the trial is shared.

### Plans for communicating important protocol amendments to relevant parties (e.g., trial participants, ethical committees) {25}

All protocol amendments need to be approved by the Committee on Health Research Ethics of the Capital Region Denmark. Deviations from the published protocol will be documented on the trial registration on ClinicalTrials.gov (NCT04902066).

### Dissemination plans {31a}

Study results will be disseminated to the scientific audience, the public, and trial participants, who through their written consent have expressed a desire to be informed. Discussions regarding the study and its results will be shared in journal publications, conference presentations, and clinical interest groups but also importantly in relevant patient interest groups.

## Discussion

To the best of our knowledge, this will be the largest RCT comparing therapist-guided VR-CBT with standard therapist-guided CBT for paranoid ideations. Initial evidence has revealed VR-CBT to be superior to waitlist control and unguided VR exposure [21, 22]. This trial will further advance knowledge in the field by comparing VR-CBT with the current gold standard of psychotherapeutic treatments for paranoid ideations, which is standard, symptom-specific CBT. While standard, symptom-specific CBT may significantly improve outcomes for paranoid ideations [19], we believe that a more efficient behavioral component, which VR-CBT is believed to generate, is essential in therapy to achieve a greater treatment effect and secondly improve cost-effectiveness. Notably, VR exposure allows for capturing the momentary experiences (thoughts, emotions, and actions) that most closely reflect the real-life situations that trigger paranoid thoughts. This opens for far-reaching possibilities in therapy perhaps not yet fully utilized.

While preliminary evidence exists on the effect of VR-CBT in established psychotic disorders [22], this will be the first RCT to evaluate the effect of VR-CBT for patients with schizotypal disorder displaying less formalized paranoid ideas (e.g., ideas of social reference). Hence, the trial will provide important evidence on the efficacy of VR-CBT for a wider group of patients with psychosis spectrum disorders shedding light on the applicability of therapy to a larger target group.

Additionally, our study clarifies potential mediators and moderators of treatment response that may aid in further identifying the clinical target group for this intervention. The assessment battery in the trial comprises several social cognitive tasks tapping different aspects of social cognition. The trial will therefore add knowledge of the potential cognitive benefits of this treatment [56]. Finally, qualitative analyses are planned which may elucidate possible mechanisms of change along with the efficacy of the specific elements in the VR-CBT protocol (e.g., use of worst-case scenarios, more realistic role-plays, and replaying the same situation but introducing alternative thoughts and behavior)

In sum, our trial will provide highly needed knowledge on the efficacy, cost-effectiveness, and acceptability of VR-CBT for paranoid ideations that may inform clinical practice.

## Trial status

Study protocol, revised version for submission, 25th of July, 2022.

Initial release 9th of April 2021.

Approximately 105-110 participants are included in our study at time of submission. 256 are to be included in total.

Approximate date when recruitment will be completed is the end of September 2023.

## Data Availability

The datasets from the study will be available from the corresponding author on reasonable request.

## Abbreviations

RCT: Randomized clinical trial
VR: Virtual reality
CBT: Cognitive behavioral therapy
VR-CBT: Virtual reality-based cognitive behavioral therapy
TAU: Treatment as usual
GPTS: Green Paranoid Thought Scale
SIAS: Social Interaction Anxiety Scale
PSP: Personal and Social Performance scale
SBQ: Safety Behavior Questionnaire
ERT: Emotion Recognition Task
SAPS: Scale of Assessment of Positive Symptoms
BNSS: Brief Negative Symptom Scale
CDSS: Calgary Depression Symptom Scale
SIDAS: Suicidal Ideation Attribution Scale
COGDIS: Cognitive Disturbance scale
DACOBS: Davos Assessment of Cognitive Biases Scale
GSE: General Self-Efficacy scale
IBT: Intentionality Bias Task
SSPA: Social Skills Performance Assessment
TALE: Trauma And Life Events checklist
R-GPTS: Revised Green Paranoid Thought Scale
CSQ: Client Satisfaction Questionnaire
SD: Standard deviations
PI: Principal Investigator
PMG: Project Management Group
DMC: Data Monitoring Comittee
